# Plant phosphate nutrition: sensing the stress

**DOI:** 10.1007/s44154-022-00039-0

**Published:** 2022-03-03

**Authors:** Viswanathan Satheesh, Ayesha Tahir, Jinkai Li, Mingguang Lei

**Affiliations:** 1 Shanghai Center for Plant Stress Biology, CAS Center for Excellence in Molecular Plant Sciences, Chinese Academy of Sciences, Shanghai, 201602 China; 2 Department of Biosciences, COMSATS University Islamabad, Park Road, Islamabad, Pakistan; 3 University of Chinese Academy of Sciences, Beijing, 100049 China

**Keywords:** Phosphate signaling, Root system architecture, Inositol pyrophosphates, InsP8, SPX domain, Plant, Human

## Abstract

Phosphorus (P) is obtained by plants as phosphate (Pi) from the soil and low Pi levels affects plant growth and development. Adaptation to low Pi condition entails sensing internal and external Pi levels and translating those signals to molecular and morphophysiological changes in the plant. In this review, we present findings related to local and systemin Pi sensing with focus the molecular mechanisms behind root system architectural changes and the impact of hormones and epigenetic mechanisms affecting those changes. We also present some of the recent advances in the Pi sensing and signaling mechanisms focusing on inositol pyrophosphate InsP_8_ and its interaction with SPX domain proteins to regulate the activity of the central regulator of the Pi starvation response, PHR.

## Introduction

Phosphorus (P), the second most important component in plant nutrition after nitrogen, is not readily available for plants. It can be bound by calcium in alkaline soils, complexed into charged aluminum and iron oxides in acidic soils, or immobilized by organic material (Kochian et al. 2004; Seguel et al. 2013). Plants absorb P predominately in the form of inorganic phosphate (Pi) (H2PO^−^ _4_/HPO_4_^− 2^) (Marschner, 2012). In certain conditions, available P in soil solution can be as low as 10 μM (Holford, 1997; do Nascimento et al. 2015). To adapt themselves to fluctuating external and intracellular levels of Pi, plants have developed complex signaling pathways. Even though plants have mechanisms in place to adapt to low Pi condition in the soil, they are not robust enough to prevent the significant yield losses that can occur particularly in agriculturally important crops. Therefore, a deeper understanding of how plants respond to Pi starvation is in order. Extensive work has been carried out in Arabidopsis, and more in rice recently, to decipher the intricate details of the Pi starvation response (PSR) (Wu et al. 2013; López-Arredondo et al. 2014). In this review, we discuss how plants respond to Pi starvation locally as well as systemically. Under local response, we discuss the importance of root system architectural (RSA) changes, the significance of hormones and the recent advances in understanding the epigenetic component driving these responses. Under systemic response, we discuss the signaling mechanism with the inositol pyrophosphates InsP_8_ and the SPX domain proteins at the core along with PHR1, the central regulator of PSR. Furthermore, a brief account of the Pi responses in yeast, and a comparison of the signaling mechanisms in plants and human is presented.

### Root system architecture remodeling

Local signals trigger adaptations of the root system architecture (RSA) such as inhibition of primary root growth and increased lateral root development in response to low external Pi to augment Pi absorption. These root developmental changes are likely governed in the rhizosphere by mechanisms that are localized to the root, as opposed to systemic sensing wherein alterations in the expression of genes involved in Pi transport and distribution, and sensors such as the SPX domain genes are triggered (Chiou and Lin, 2011; López-Arredondo et al. 2014; Wild et al. 2016; Dong et al. 2019; Zhu et al. 2019). A significant amount of work has been reported on RSA changes in Arabidopsis and this section focuses on research emanating from this model plant. The genetic interaction of the ferroxidases LOW PHOSPHATE ROOT1/2 (LPR1/2), which are expressed in the root tip (meristem and root cap), with PHOSPHATE DEFICIENCY RESPONSE2 (PDR2) under Pi-depleted condition plays a significant role in the inhibition of primary root (PR) growth as part of the local Pi sensing mechanism (Svistoonoff et al., 2007; Ticconi et al. 2009). The endoplasmic reticulum (ER)-located PDR2, a P5-type ATPase, functions as a Pi-dependent checkpoint in root development by controlling SCARECROW (SCR) expression in Pi-depleted root, which affects root patterning and stem-cell maintenance (Ticconi et al. 2009). It is likely that LPR1 is trafficked from the ER to the plasma membrane to act as a cell wall-associated ferroxidase that oxidizes Fe^2+^ to Fe^3+^ under Pi-depleted condition. In a parallel pathway, the SENSITIVE TO PROTON RHIZOTOXICITY (STOP1) transcription factor activates the ALUMINIUM ACTIVATED MALATE TRANSPORTER 1 (ALMT1), which is integrated into the plasma membrane where it functions as a malate secretion system into the apoplasm (Müller et al. 2015; Mora-Macías et al. 2017; Balzergue et al. 2017; Godon et al. 2019). LPR1-mediated oxidation of Fe^2+^ leads to accumulation of Fe^3+^ in the apoplasm, which in complex with malate activates ROS production that promotes callose deposition in the stem cell niche of the PR. Callose deposition hampers trafficking of key transcription factors such as SHR resulting in the loss of stem cell function and inhibition of primary root growth (Müller et al. 2015). A recent study has shown that STOP1 has a broader role to play as ammonium uptake by the AMT1 transporters under Pi starvation triggers rhizosphere acidification of the root surface, which results in the exudation of organic acids due to the accumulation of STOP1 (Tian et al. 2021). Presence of organic acids improves Pi acquisition from insoluble P sources. Also, this study demonstrates that ammonium acts upstream of STOP1 leading to Fe-malate accumulation inhibiting primary root growth in conjunction with LPR1 and LPR2 ferroxidases. The CLAVATA3 (CLV3)/ENDOSPERM SURROUNDING REGION14 (CLE14) peptide is induced by Pi starvation acting downstream of the PDR2–LPR1 module and its expression pattern overlaps Fe distribution in the root apical meristem under Pi-deficient condition (Gutierrez-Alanis et al., 2017). CLV2/PEP1 RECEPTOR2 (PEPR2) receptors perceive CLE14 at the plasma membrane leading to suppress the SHR–SCARECROW (SCR) and PIN-FORMED (PIN)–auxin pathways leading to callose-independent RAM exhaustion (Gutierrez-Alanis et al., 2017). These developments, therefore, underline the importance of the primary root in perceiving Pi levels and in triggering the response mechanisms that ultimately result in the reprogramming of the RSA.

Interestingly, blue light is essential, and may be enough, for primary root growth inhibition under Pi deficiency (Zheng et al. 2019). Blue light can activate the malate-mediated photo-Fenton reaction and hydroxyl radicals are produced by a Fe redox cycle, which is formed by a canonical Fenton reaction in the apoplast. The hydroxyl radicals thus formed cause primary root growth inhibition (Zheng et al. 2019). These observations were further confirmed recently when some of the molecular players involved in root growth inhibition were revealed (Gao et al. 2021). Cryptochromes and other downstream signaling components such as SPA1, COP1 and HY5 play major roles in RSA modulation. Blue light is required for the translocation of shoot-derived HY5 to the root to autoactivate root HY5, which activates *LPR1* to promote primary root inhibition under Pi starvation. For a more in-depth view of the developmental responses of the root under Pi deficiency, the readers are referred to the excellent review by Professor Dong Liu (Liu, 2021).

### Hormonal control

One of the most important hormones that is implicated in the responses of RSA to Pi starvation is auxin. On Pi starvation, the root tip and lateral root primordia are sites of increased auxin signaling resulting in primary root growth inhibition and enhanced formation of lateral roots (Lopez-Bucio et al., 2002; Nacry et al. 2005) and the changes in RSA could be attributed to auxin redistribution in the roots (Nacry et al. 2005). Exogenous application of auxin showed that Pi-starved plants were more sensitive than plants with adequate Pi supply, and the observed sensitivity is dependent on the auxin receptor TIR1 and the transcription factor ARF19 (Lopez-Bucio et al., 2002; Perez-Torres et al., 2008). For lateral root initiation, as both auxin and TIR1-dependent auxin signaling are important, and the fact that TIR1 is a target of PHR1, it is reasonable to assume a link between lateral root formation, auxin signaling and Pi starvation (Castrillo et al. 2017; Du and Scheres, 2018). Apart from TIR1, genes from the Aux/IAA and ARF gene families are also putative targets of PHR1 (Castrillo et al. 2017). Genes such as ARF7, ARF19 and SLR/IAA14 are involved in the Pi starvation signaling pathway in both Arabidopsis and rice and are required for the regulation of lateral root initiation (Okushima et al. 2005; Narise et al. 2010; Shen et al. 2013; Huang et al. 2018). Other genes implicated in Pi-driven auxin sensitivity are the Arabidopsis Receptor Kinase2 (ARK2)-Ubox/armadillo repeat-containing E3 ligase9 (PUB9), rice *OsARF12*, *OsARF16*, and *OsPht1;8* genes, and cotton GbWRKYxu1 (Deb et al. 2014; Shen et al., 2013, Wang et al. 2014a, Jia et al. 2017; Xu et al. 2012). Under low Pi condition, auxin-dependent root hair growth is triggered by the transport of auxin from the root apex to differentiation zone with the help of TRYPTOPHAN AMINOTRANSFERASE OF ARABIDOPSIS1 (TAA1, auxin synthesis) and AUX1 (auxin transport) (Bhosale et al. 2018). Furthermore, in trichoblasts with elevated auxin levels, auxin induces the transcription of transcription factors ARF19, ROOT HAIR DEFECTIVE 6-LIKE2 (RSL2), and RSL4, which mediate a gene expression cascade promoting root hair elongation for improved Pi foraging (Bhosale et al. 2018). In a parallel study, the authors also conclude from studies in rice that auxin and OsAUX1 are important in augmenting root foraging for Pi in a mechanism similar to Arabidopsis (Giri et al. 2018).

Ethylene (ET), cytokinin (CK), gibbberellin (GA), strigolactone (SL) and jasmonate (JA) are other hormones that play significant roles in integrating environmental cues and RSA. ET is a positive regulator of root hair growth and root elongation under Pi starvation (Borch et al. 1999; Ma et al. 2003; Zhang et al. 2003). When extracellular Pi is low, ET biosynthesis is enhanced and ETHYLENE-INSENSITIVE3 (EIN3), a key transcription factor in ET signalling, interacts with the promoters of genes targeted by RSL4 (Song et al. 2016). RSL4 being an important factor in root hair development, along with its homologs, further enhances their expression levels resulting in increased root hair formation (Song et al. 2016). Additionally, EIN3, in complex with FHY3, FAR1 and HY5, binds to the PHR1 promoter to regulate its expression and drive PSRs (Liu et al. 2017). The genes ACS2, ACS4, ACS6 and ACO encoding for enzymes necessary for the conversion of AdoMet to ACC (1-aminocyclopropane-1-carboxylate) and ACC to ethylene, are induced under Pi starvation in Arabidopsis (Morcuende et al. 2007; Thibaud et al. 2010; Lei et al. 2011). CK is critical in balancing the root:shoot ratio, and under Pi starvation, CK increases root:shoot ratio, as root growth is favored over shoot and CK concentrations are reduced under Pi starvation (Kuiper and SteingrÖVer, 1991; Franco-Zorrilla et al. 2002). Pi starvation-inducible genes are also deregulated by CK leading to an increase in Pi concentration (Martin et al. 2000; Franco-Zorrilla et al. 2002; Wang et al. 2006). Among the different types of CK, trans-zeatin is reduced under Pi starvation while cis-zeatin and cis-zeatin roboside are increased significantly (Silva-Navas et al. 2019). The two latter types are PHR1-dependent and cis-zeatin regulates genes involved in cell growth and root hair elongation, and both cause an increase in Pi concentrations in roots. Interestingly, root growth and lateral root formation is enhanced when the cis-zeatin:trans-zeatin ratio is higher, and cis-zeatin is essential for root hair elongation and Pi allocation to the root and shoot under low Pi condition (Silva-Navas et al. 2019).

SLs are plant metabolites that are derived from carotenoids and are synthesized mainly in the root (Yoneyama et al. 2013; Al-Babili and Bouwmeester 2015; Lopez-Obando et al. 2015). Under Pi starvation, SLs inhibit shoot branching and regulates important RSA changes such as lateral root formation and root hair density (Umehara et al. 2008, Kapulnik et al. 2011). Not only do the SLs move acropetally as a systemic signal while controlling shoot branching under Pi-depleted condition, they can also be synthesized in the shoot to control branching (Beveridge et al. 1997; Foo et al. 2001; Sorefan et al. 2003). Auxin-mediated control of RSA could also involve GA, and the core components of the GA signaling pathway, the DELLA proteins, contribute to anthocyanin accumulation and RSA changes and are not likely to be involved in the expression of PSI genes or Pi uptake (Jiang et al. 2007). However, a later study showed that the transcription factor MYB62 could regulate PSR through alterations in GA metabolism and signaling (Devaiah et al. 2009). JA is known to inhibit root growth and specifically controls lateral root growth and root hair development in Arabidopsis and rice (Cai et al. 2014; Wang et al. 2002). A recent study showed that overexpression of the Pi-inducible *OsJAZ11* reduced the PSR under Pi-depleted condition along with increased primary add seminal root elongation (Pandey et al. 2021). *OsJAZ11* is transcriptionally regulated and, interestingly, also physically interacts with OsSPX1. VIH2 has been shown to regulate the synthesis of inositol pyrophosphate InsP_8_ and JA-dependent defense response in Arabidopsis, and InsP_8_ has been implicated as a signaling molecule in driving PSR in Arabidopsis (Laha et al. 2015; Dong et al. 2019; Zhu et al. 2019). Though studies on the role of JA in Pi sensing are few and far between, with these recent developments, several more studies are expected.

### Epigenetic control

Gene regulation in plants under Pi starvation is well studied at the transcriptional and posttranscriptional levels (López-Arredondo et al. 2014). Studies in Arabidopsis and rice have shown that for proper PSR, epigenetic control as an added level of regulation is essential (Yong-Villalobos et al. 2015; Secco et al. 2015; Table 1). Yong-Villalobos and co-workers showed that in the Arabidopsis *drm1*, *drm2*, and *cmt3* single mutants the *drm1 drm2 cmt3* triple mutant the primary root length was shorter than the wild-type under Pi-depleted condition. A similar observation was also made for lateral root density. Observations made in this study clearly showed that epigenetic marks, DNA methylation, are key to establishing proper morphophysiological PSR. ARP6 is a nuclear actin-related protein and in Arabidopsis, it is required for the proper deposition of H2A.Z at several PSR genes. Loss-of-function *ARP6* mutation resulted in the loss of H2A.Z at the target loci, which led to shortening of primary roots and increased number and length of root hairs (Smith et al. 2010). Further, a reduction in H2A.Z occupancy in chromatin was reduced in *atipk1–1* mutant leading to an increase in the number of Pi starvation-inducible genes (Kuo et al. 2014). Members of the histone deacetylase family, HDA6, HDA9 and HDA19, are involved in the regulation of root cell length (Chen et al. 2015). Recently, histone deacetylase 1 (HDC1), was found to affect primary root growth and LPR1/2-mediated iron deposition at the root tips under low Pi condition (Xu et al. 2020). Therefore, HDC1 is an important component of the local Pi response acting as a negative regulator of RSA changes under Pi-depleted condition. These recent studies clearly show that chromatin and DNA methylation pattern remodeling are active components of a plant’s response to Pi-depleted condition or could possibly be due to the changes that occur during the epigenetic regulation of PSR genes (Yong-Villalobos et al. 2015).

**Table 1:** Studies on epigenetic control of Pi nutrition

Epigenetic mechanism	Salient findings	Reference
H3K4me3	Involvement of Arabidopsis AL6 in root hair elongation	Chandrika et al. (2013)
Histone acetylation	HDA19 influences root cell elongation and controls expression of a subset of PSR	Chen et al. (2015)
H2A.Z	ARP6 is required for H2A.Z deposition to regulate PSR genes; also in concert with IPK1	Smith et al. (2010); Kuo et al. (2014)
DNA methylation	Arabidopsis: Genome-wide changes, either limited (Secco et al. 2015) or extensive (Yong-Villalobos et al. 2015), associated with gene expression Rice: 55% of the differentially methylated cysteines were linked to transposable elements; lack of transgenerational inheritance	Secco et al. (2015); Yong-Villalobos (2015); Secco et al. (2017)
Small RNA	Changes in DNA methylation patterns can be mediated by TE-derived phloem-mobile 24-nt sRNAs	Zhang et al. (2016); Ham and Lucas (2017)

### SPX domain proteins: the sensors

The SPX domain (Pfam PF03105) plays an increasingly important role in elucidating the mechanism of Pi homeostasis (Secco et al. 2012). This was initially identified in yeast and is named after the Suppressor of Yeast *gpa1* (Syg1), which is a negatively affected mating pheromone signal in yeast when it is truncated, a cyclin dependent kinase (CDK) inhibitor phosphatase 81 (Pho81), and the human Xenotropic and Polytropic Retrovirus receptor1(Xpr1). Xpr1 regulates polyphosphate in platelets and was implicated in thrombosis in vivo (Mailer et al. 2021), and influences intracellular ATP levels because of the repression of IPK6 (Moritoh et al. 2021). The SPX domain is quite conserved and hydrophilic, usually located at the N-terminus of eukaryotic proteins. The SPX domain can be divided into three subdomains, each containing 30–40 amino acids, with an average length of 165 amino acids. Subdomains are separated from each other by low similarity regions (Liu et al. 2018). Proteins containing the SPX domain in plants can be divided into four groups: the first group contains only the SPX domain, such as AtSPX1 in Arabidopsis (Duan et al. 2008), OsSPX1/2 (Wang et al. 2009) and OsSPX4 (Lv et al. 2014) in rice. The second is proteins containing one SPX domain and one EXS domain, such as Arabidopsis AtPHO1, which is an ortholog of the human XPR1, regulates Pi transport from the root to the shoot (Wild et al. 2016). The third type is the protein containing an SPX domain and RING domain, such as AtNLA, a ubiquitin connection containing both SPX and RING domain, requires UBC24 to mediates polyubiquitination of Pht1;4 (Park et al. 2014). The fourth category is proteins containing one SPX domain and one MFS domain, such as OsVPE1 and OsVPE2, which localize on the tonoplast and mediate Pi efflux from the vacuole into cytosol (Wang et al. 2015).

The SPX domain has been reported as a Pi sensor and SPX proteins interact with PHR homologous proteins and regulate their function by binding to them under Pi-replete condition in the presence of inositol pyrophosphates (PP-InsPs) and prevents the PHR proteins from entering the nucleus (Liu et al. 2010; Shi et al. 2014; Wang et al. 2014b; Puga et al. 2014; Lv et al. 2014; Wild et al. 2016; Dong et al. 2019; Osorio et al. 2019). Similar mechanisms have been reported in other organisms as well. In the pathogenic process of human-pathogenic fungus *Cryptococcus neoformans*, InsP_7_ synthesized by Kcs1 regulates fungal virulence by binding to a conserved lysine surface cluster in the SPX domain of Pho81 (Desmarini et al. 2020). SPX negatively regulates Pi uptake and metabolism through PSR regulator (PHR) as an intermediate in the marine phytoplankton, *Phaeodactylum tricornutum* (Zhang et al. 2021). While SPX proteins have largely been implicated in the Pi response mechanism, GmSPX5 from soybean was shown to play a key role in nodule adaptation to low Pi condition (Zhuang et al. 2021) adding a new dimension to the SPX protein function.

### Inositol pyrophosphates: the signaling molecules

**Fig. 1:**
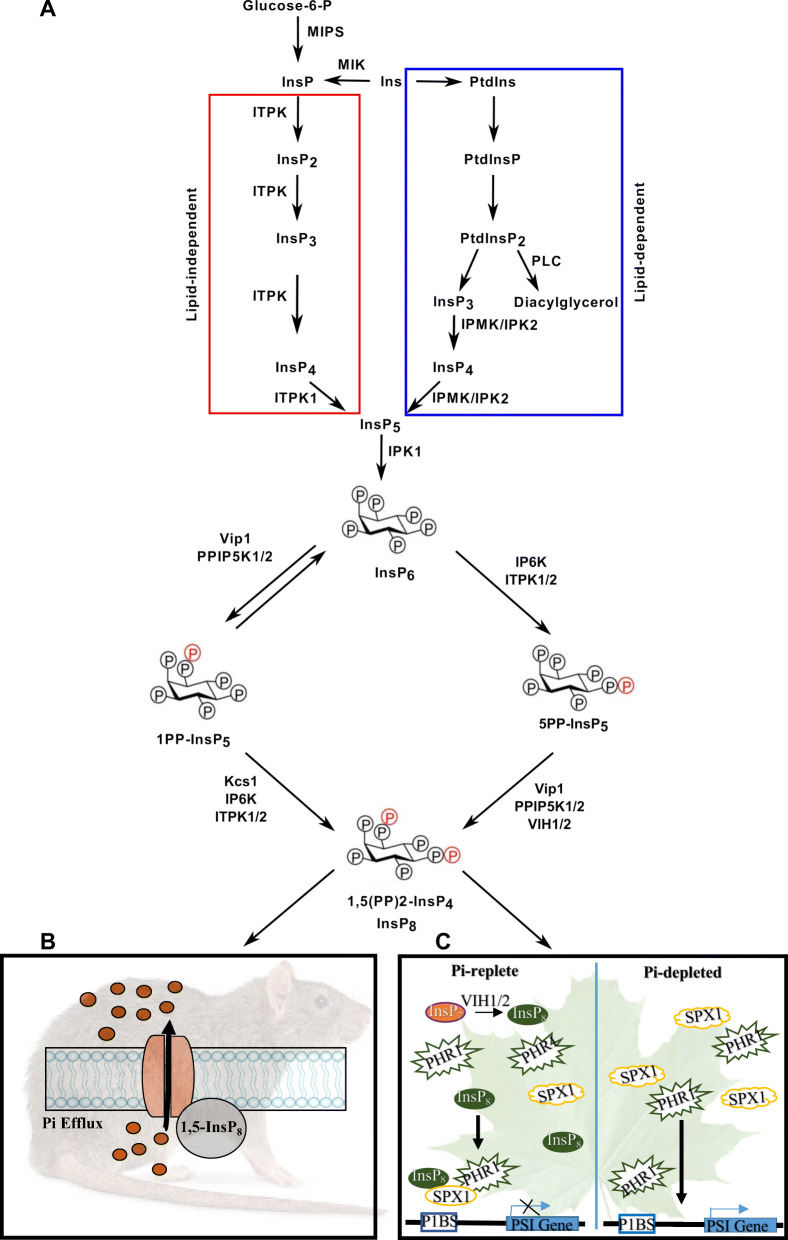
Biosynthesis pathways of PP-InsP and IP8 signalling in plants and humans. **A** Schematic model of InsP and PP-InsP biosynthesis pathway in humans (via Lipid-dependent pathway) and plants (via Lipid-dependent and -independent pathways). Key enzymes in InsP and PP-InsP pathways are shown for each reaction. **B** Pi sensing and InsP_8_ signaling in human/animal system is presented. **C** Pi sensing and InsP_8_ signalling under Pi-replete and Pi-depleted conditions are presented

The PP-InsPs are novel and unique signaling molecules that are implicated in a wide variety of biological processes in eukryotes (Azevedo and Saiardi 2017; Shears 2018; Lorenzo-Orts et al. 2020). PP-InsPs are high-energy pyrophosphate containing molecules obtained by the additional phosphorylation of inositol polyphosphates. The inositol polyphosphate InsP_6_, is considered a storage molecule, and is accumulated in the form of phytic acid particularly in the seeds (Secco et al. 2017). From InsP_6_, phosphorylation at positions 5 leads to the formation of PP-InsP, InsP_7_, catalyzed by the enzyme inositol phosphate kinase ITPK1(Riemer et al. 2021). InsP_7_ is then converted to InsP_8_ by VIH1 and VIH2, and these PP-InsPs have been reported to act as signaling molecules in plants (Laha et al. 2015; Wild et al. 2016; Dong et al. 2019; Zhu et al. 2019; Fig. 1A).

### InsP_8_-SPX- PHR: the interaction

PHR1, containing the MYB-like DNA-binding domain and a coiled-coil domain, belongs to the GARP transcription factor family and is homologous to the PSR1, which was shown to regulate P metabolism in *Chlamydomonas reinhardtii* (Wykoff et al. 1999; Rubio et al. 2001). PHR1 binds to an imperfect palindromic motif (5′-GNATATNC-3′) known as the P1BS element or the PHR1-binding sequence, which is found in most of the Pi starvation-inducible genes. PHL1 (PHR1-LIKE 1), a paralog of PHR1, functions redundantly to regulate the expression of a subset of PSR genes (Bustos et al. 2010). Orthologous genes of PHR1 have been identified in plants including rice (Zhou et al. 2008), maize (Wang et al. 2013a), wheat (Wang et al. 2013b) and soybean (Lu et al. 2020). As previously mentioned, PHR function is regulated by the SPX domain containing proteins, which act as cellular receptors for the PP-InsPs.

VIH1 and VIH2 are homologous to the yeast and animal Vip1/PPIP5Ks (Desai et al. 2014; Laha et al. 2015) and are bifunctional kinase/phosphatase enzymes (Zhu et al. 2019). Previous studies have suggested that the preferred ligand for SPX proteins is InsP_8_ as the *vih1 vih2* double mutants show severe defects in growth phenotype and Pi signaling, and under in vivo conditions, InsP_8_ restores the interaction between SPX1 and PHR1 better than 5-InsP_7_ (Dong et al. 2019; Zhu et al. 2019; Ried et al. 2021). The 5-InsP_7_ isomer, the precursor of InsP_8_, is generated by ITPK1, the InsP_6_ kinase in planta (Laha et al. 2019). We along with others showed that InsP_7_ was phosphorylated by VIH1 and VIH2 to generate InsP_8_ (Laha et al. 2015; Dong et al. 2019; Zhu et al. 2019). A recent study has shown that under Pi starvation 5-InsP_7_ and 1/3-InsP_7_ were also reduced, and recovered on Pi resupply (Riemer et al. 2021). Riemer and co-workers also showed that synthesis of 5-InsP_7_ and InsP_8_ in planta is Pi-dependent mediated by ITPK1. The authors also provide genetic evidence for the interdependence of ITPK1 and VIH2 for the maintenance of Pi homeostasis in plants. In the context of VIH1 and VIH2, under Pi-replete condition phosphatase activity is allosterically regulated by Pi resulting in an increase in InsP_8_ level culminating in the formation of a InsP_8_-SPX- PHR complex (Gu et al., 2017a; Zhu et al. 2019). Kinase activity is reduced under Pi-deficient condition as ATP levels drop leading to a decrease in the levels of InsP_8_ and the complex breaks down (Dong et al. 2019; Zhu et al. 2019). The released PHR can now transactivate the PSR genes by binding to the P1BS motif as a dimer (Rubio et al. 2001). Recent work has shown that the CC domain of PHRs is targeted by the InsP_8_-SPX1 complex and regulates the promoter binding ability of PHRs as dimers through their SPX receptors (Ried et al. 2021). Interestingly, Zhou and co-workers (Zhou et al. 2021) show that the under Pi-replete condition, InsPs bind to the SPX protein to stabilize the helix α1 structure, which enables the InsPs to allosterically decouple the PHR protein dimer. The SPX protein is then able to interact with the MYB domain and blocks PHR protein from binding to the promoters of the PSR genes. Therefore, SPX inhibition of PHR activity occurs at two levels of oligomerization and DNA binding (Zhou et al. 2021). These recent advances in Pi signaling have opened new avenues in plant research to develop crops with higher Pi use efficiency.

### Pi sensing: the yeast response

**Fig. 2:**
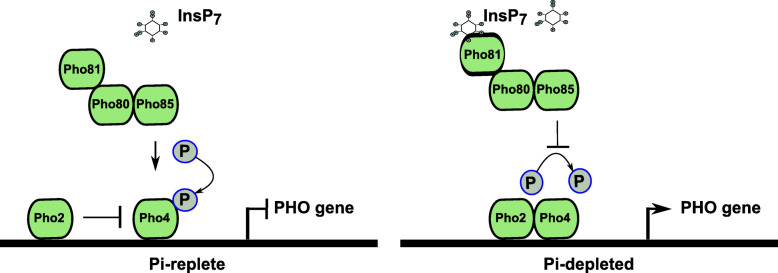
A schematic showing Pi sensing and regulation of gene expression under Pi-replete and -depleted condition in yeast. Pho81 negatively regulates the Pho80-Pho85 complex and its binding to the complex is Pi-independent. Under Pi-replete condition, Pho81-Pho80-Pho85 complex phosphorylates Pho4 and it cannot activate the PHO genes. Under Pi-depleted condition, InsP_7_ interacts allosterically with the Pho81-Pho80-Pho85 tertiary complex preventing Pho4 from being phosphorylated by the complex. Lack of phosphorylation allows Pho4 to activate the genes in the PHO pathway

Pi sensing in yeast is a well-studied mechanism and in *Saccharomyces cerevisiae*, the Pi signaling pathway (also called the PHO pathway) is activated under Pi starvation and several genes that are involved in the uptake and storage of Pi are upregulated (Bergwitz and Jüppner, 2010; Secco et al. 2012; Sabbagh, 2013; Sengottaiyan et al., 2011; Conrad et al. 2014). Pho4, along with its coactivator Pho2, is a key factor in mediating PSR in yeast by regulating the expression of the Pi-responsive genes (Magbanua et al. 1997a; Fig. 2). In fact, the Pho4-Pho2 interaction enhances the affinity for Pho4-binding sites in the promoters of PHO genes (Shao et al. 1996; Magbanua et al. 1997a, b). Pho4 activity and subcellular localization is determined by the phosphorylation of its serine residues by the cyclin-dependent kinase complex Pho80-Pho85 (Toh-e et al. 1988; Kaffman et al. 1994; O’Neill et al. 1996). As a requisite transcription factor, it is dephosphorylated and accumulated in the nucleus to induce the expression of Pho4-dependent PHO genes (Zhou and O’Shea 2011).

The cyclin-dependent kinase inhibitor Pho81 plays a key role in the PHO pathway as a negative regulator of the Pho80-Pho85 complex by binding to it independent of Pi availability and inhibits kinase activity only under Pi-limiting condition (Schneider et al. 1994; Ogawa et al. 1995; O’Neill et al. 1996; Huang et al. 2001). A conformational change is induced in Pho81 by the allosteric interaction of PP-InsPs with the Pho81-Pho85-Pho80 tertiary complex, and this change prevents Pho4 from accessing the Pho85-Pho80 kinase active site (Lee et al. 2007; Lee et al. 2008). Unphosphorylated Pho4 enters the nucleus and activates the transporters (Fig. 2).

There are five transporters in yeast viz., Pho84, Pho87, Pho89, Pho90 and Pho91. While Pho84 and Pho89 are high-affinity transporters, Pho87, Pho90 and Pho91 are low affinity transporters. Under low internal Pi, Pho84 is activated and there is an increase in Pi uptake generating a negative-feedback loop. Interestingly, *Spl2* is also upregulated, which negatively regulates Pho87 and Pho90 low-affinity transporters (Wykoff et al. 2007). Levy et al. (2011) have shown that a dual-transporter system with high- and low-affinity transporters, could extend the preparation for starvation and enables yeast cells to recover subsequently. Therefore, as Pi concentration depletes gradually in the environment, the transition of yeast cells from Pi-replete to Pi-depleted condition is longer, and the cells can better adapt themselves to Pi deprivation making recovery more effective on resupplying Pi. Among the transporters, Pho84 acts as a Pi sensor and transporter, and on resupplying Pi after prolonged starvation it is internalized from the plasma membrane and degraded. Pho4 is phosphorylated on Pho84 degradation after Pi resupply by Pho80-Pho85, and exported to the cytoplasm and the PHO genes are repressed (Giots et al. 2003; Mouillon and Persson 2006; Samyn et al. 2016).

The PHO pathway is also involved in the synthesis of polyphosphates, which depends on the Vacuolar Transport Chaperone (Vtc) complex in yeast. This comprises Vtc1, Vtc2, Vtc3, and Vtc4, where Vtc4 is the catalytic subunit (Hothorn et al. 2009). Vtc1/2/4 and Vtc1/3/4 are two different Vtc complexes found in the endoplasmic reticulum and vacuole, respectively, under Pi-replete condition, and both complexes are found in the vacuole under Pi-depleted condition (Hothorn 2009). Among the various PP-InsPs, 5-InsP_7_ regulates VTC in vivo (Gerasimaite et al., 2017). VTC synthesizes InsPs under Pi-replete condition, and the cells use the reserve stocks under Pi-depleted condition. Higher activity of VTC leads to Pi depletion in the cells triggering the PHO pathway under Pi-replete condition (Desfougères et al. 2016). Thus the yeast Pi starvation response mechanism shares similarities with that found in the plant systems.

### Plant and human InsP_8_ synthesis and Pi sensing: the commonalities

Inositol phosphates (InsPs) are gaining attention for broadening our understanding of energy metabolism and nutrient sensing in humans as well as plants (Chakraborty et al., 2011; Hatch and York 2010). Although they play conserved functions in various eukaryotic cellular processes, their biosynthesis and their function in Pi sensing (the most common and widely known attribute of this family) (Lee et al. 2020), differs between humans and plants with a few exceptions detailed in this section.

Biosynthesis of bioactive inositols (inositol polyphosphates) can be via lipid-dependent or lipid-independent pathway (Fig. 1A). Lipid-dependent pathway is shared between animals and plants where initiation involves phosphorylation of phosphatidylinositols (PtdIns) (Berridge and Irvine 1989; Stevenson-Paulik et al. 2002). Phospholipase C (PLC) is a conserved enzyme in humans and plants for conversion of phosphatidylinositol 4,5 bisphosphate (PtdIns(4,5)P2) into diacylglycerol (DAG) and Ins(1,4,5)P3. This initiation process is carried out in the plasmamembrane and thereafter Ins(1,4,5)P3 is released into the cytosol where successive addition of phosphates is mediated by Inositol Polyphosphate Multikinases (IPMK) in animals, and by the Inositol Pentakisphosphate 2-Kinase (IPK2) in plants (Berridge and Irvine 1989; Lee et al., 2020; Fig. 1).

Lipid-independent pathway for InsP_6_ synthesis has been characterized well for plants compared to animals. In the first step of the pathway, myo-inositol kinase (MIK) was reported to be involved in phosphorylation of inositol at 3 position to form InsP (Kim and Tai 2011; Shi et al. 2005). Multiple studies showed that MIK gene functionality is almost conserved in all the species of plants (Loewus and Murthy, 2000; Shi et al. 2005). Contrarily, in humans, MIK ortholog with only limited homologous regions were observed for one ribokinase as well as for various adenokinase genes. Hence, either human MIK gene is distinct compared to that of plants or they do not require MIK gene as myo-inositol phosphate synthase (MIPS) catalyzes the reaction of glucose-6-phosphate conversion to Inositol 3-phosphate (Funkhouser and Loewus 1975). Similar to MIK, another enzyme LPA1 (Low Phytic Acid) involved in the formation of InsP_2_ from InsP in plants also lacks a definitive ortholog in the human genome. Recently, Desfougères and colleagues reported that plants and animals both have Inositol 1,3,4-Trisphosphate 5/6-Kinases (ITPKs) to avoid the need for LPA1 enyzme for final product synthesis (Desfougères et al. 2019). Finally, the conversion of InsP_5_ to InsP_6_ requires IPK1 in plants and its counterpart in humans is HsIPPK (Kuo et al. 2018; Park et al. 2018). Inositol hexakisphosphate (InsP_6_) is the major source of Pi storage in seeds. Owing to Multidrug Resistance Protein 5 (MRP5) which activates InsP_6_ transport to the vacuolar membrane, InsP_6_ can be stored in higher amounts in plants (Freed et al. 2020; Raboy 2001).

Pyrophosphorylation of InsP_6_ leads to the synthesis of PP-InsPs, which contain highly energetic phosphoanhydride bonds. As mentioned earlier, InsP_6_ can synthesize 1,5-InsP_8_ via 1-InsP_7_ or 5-InsP_7_ intermediates, with 5-InsP_7_ being the major route of InsP_8_ synthesis. Previously, PPIP5Ks (VIH/VIP in plants) were assumed to inter-convert InsP_6_ to InsP_7_ and InsP_7_ to InsP_8_ in two successive steps (Campo and San Segundo 2020; Chakraborty et al. 2011). Recently published work provided evidence that ITPKs are the responsible kinases for InsP_6_ conversion to InsP_7_ in plants in lieu of PPIP5Ks, whereas in humans this step involves IP6Ks (Adepoju et al., 2019). PPIP5Ks are mainly involved in the interconversion of 1-InsP_7_ to 1,5-InsP_8_ and as mentioned above this route is the major source of InsP_8_ synthesis. These kinases are attracting attention for various therapeutics in humans and also for stress alleviation in plants (Dollins et al. 2020; Gu et al. 2016).

PPIP5Ks present conserved structures in both humans and plants with KD (N-terminal ATP-grasp kinase domain), PD (C-terminal phosphatase domain) and PH (Pleckstrin-homology) domains. Despite limited sequence identity of AtITPKs and PPIP5Ks, KD domain is conserved in these two kinases (Wang et al. 2012, 2014c). PH domains are common among signaling proteins and have affinity for phospholipids and their derived head groups. Arginine residue is required for ligand binding in human PH domain of PPIP5Ks whereas in Arabidopsis, the purpose is served by a lysine residue (Gokhale et al. 2011). Moreover, AtPIPP5Ks and HsPIPP5Ks also share a C-terminal intrinsically disordered region, crucial for protein-protein interactions (Machkalyan et al. 2016).

PD-KD domains render unique features to PPIP5Ks and their balanced activities are critical for growth, development and Pi sensing in plants (Zhu et al. 2019). Under certain conditions in humans, PD can restrict the KD activity and PD mutation of HsPIPP5K resulted in autosomal recessive non-syndromic hearing loss. Hence, the importance of PD-KD balance in humans was demonstrated and this balance can be disrupted by Pi, which is a strong inhibitor of PD domain in one of HsPPIP5K2 isoform (Gu et al., 2017a). Briefly, PPIP5Ks activity is affected by external Pi in humans. In plants, this is not proven yet as the PD domain of PPIP5Ks is recalcitrant to enzymatic observations. However, in plants AtPPIP5K loss-of-function mutations attributed to increased InsP_7_ and decreased InsP_8_ levels with stunted growth and Pi sensing defects (Dong et al. 2019; Zhu et al. 2019).

As discussed above, InsPs and PP-InsPs synthesis pathways exhibit similarities and differences among humans and plants at various levels with common downstream InsP_8_ synthesis (Fig. 1B, C). InsP_8_ is a proven metabolic messenger of intra-cellular Pi status across humans and plants (Dong et al. 2019; Li et al. 2020). Yet, this signaling pathway also differs in terms of enzymatic players in both kingdoms (Lee et al. 2020). Among all eukaryotes PP-InsP-SPX interaction is a crucial signaling module for the control of cellular Pi homeostasis. SPX domain contains 135–380 amino acids and there are four groups of SPX domain-containing proteins in plants (Secco et al. 2012; Wang et al. 2021). Contrarily, in the human genome, the only SPX domain-containing protein with cellular Pi efflux control is the Xenotropic and Polytropic Retrovirus Receptor 1 (XPR1).

Prior to the Wild et al. study, the SPX domain was considered to have a strong binding affinity directly to Pi. This report changed this notion and showed higher binding affinity of the SPX domain to InsP and PP-InsP compared to Pi and PPi (Wild et al. 2016). In plants, under Pi-sufficient condition, PP-InsPs expedite SPX interaction with PHR1 and its homologs to prevent PSR genes expression. From available data, it is safe to assume that high cellular Pi concentrations led to VIH-mediated InsP_8_ synthesis, which eventually facilitated SPX-PHR1 binding and finally transcription of PSR genes is turned off. On the other hand, low cellular Pi leads to a decrease in InsP_8_ levels and PHR1 is available for the activation of PSR genes (Dong et al. 2019; Zhu et al. 2019).

In earlier studies on human cells, cellular InsP_7_ as well as InsP_8_ levels were considered to be correlated with levels of cellular Pi (Lonetti et al. 2011; Gu et al., 2017b). Recent genetic studies with PPIPK5s knock out mutants in humans alleviated XPR- mediated Pi efflux by reduction in InsP_8_ levels. Restoration of Pi efflux resulted in Pi efflux rescue, hence, highlighting InsP_8_ as the main cellular Pi-sensing signal (Li et al. 2020). Therefore, studies from humans and plants argue that InsP_8_ is the ligand that modulates SPX domain interaction which in turn controls PSR gene expression by interacting with a transcription factor or controls Pi efflux directly in humans.

### Challenges and perspectives

PSR is multifaceted. Therefore, understanding the mechanisms and making use of the knowledge gained in addressing problems of nutrient stress necessitates a multifaceted approach. With significant progress being made in Pi signaling *vis-a-vis* the signaling molecules, PP-InsPs, and the sensor, SPX domain, the diversity of SPX domain-containing proteins in plants need to be explored further as presence of an expanded SPX family compared to humans would help define other functions in this sessile group of organisms. Similarly, PP-InsP-SPX interaction should be characterized to define interaction components of other SPX-domain containing protein members. Another challenge is whether a single enzyme should be targeted in PP-InP biosynthesis pathway or PP-InP interactions with other proteins should be focused to have a better view of PP-InsP targets and their interacting mechanisms (Furkert et al. 2020; Lee et al. 2021). Moreover, a deeper understanding of these mechanisms would be beneficial for the development of new therapeutics in human disease as well as in understanding plant mechanisms related to these PP-InsP interactions. In recent times, work on epigenetic regulation in PSR is gathering momentum. Reduced cost of sequencing has enabled large-scale sequencing studies particularly on DNA methylation. Since most work has focused on Arabidopsis and/or rice to a large extent, epigenomic studies should be extended to other crops as well and better analysis methods are required to obtain a better understanding of the mechanisms that drive epigenetic changes in crops under low Pi stress. This would require concerted efforts as most of the breakthrough studies are still being made in Arabidopsis and it is difficult to fathom how it would translate to other crops, and in particular, how knowledge on these discovered mechanisms would translate under field conditions, which is a completely different ecosystem compared to controlled laboratory conditions. The other aspect of utilizing epigenetic mechanisms in plant breeding efforts is the need to understand transgenerational inheritance of the epigenetic marks. Therefore, a thorough understanding mechanisms that underlie low Pi-stress resilience is required to develop crops that are tolerant to such nutrient stress by engineering the epigenome.

## Data Availability

Not applicable.

## Abbreviations

P: Phosphorus
Pi: Phosphate
PSR: Phosphate stress response
RSA: Root system architecture
Fe: Iron
ET: Ethylene
CK: Cytokinin
GA: Gibberellin
SL: Strigolactone
JA: Jasmonate
InsP: Inositol polyphosphates
PP-InsP: Inositol pyrophosphates
TF: Transcription factor
